# Impact of a multi-disciplinary team-based care model for patients living with diabetes on health outcomes: a mixed-methods study

**DOI:** 10.1186/s12913-024-11062-4

**Published:** 2024-06-18

**Authors:** Jacquelyn Jacobs, Alyn Dougherty, Banita McCarn, Nazia S. Saiyed, Stacy Ignoffo, Christina Wagener, Cindy San Miguel, Linda Martinez

**Affiliations:** 1 Sinai Urban Health Institute, 1500 South Fairfield Avenue, Chicago, IL 60608 USA; 2 Sinai Chicago, 1500 South Fairfield Avenue, Chicago, IL 60608 USA

**Keywords:** Diabetes, Team-based care, Multi-disciplinary team, Implementation science, Participatory research, Endocrinology

## Abstract

**Background:**

Individuals facing socioeconomic hardship experience higher than average rates of chronic disease, such as diabetes, with less access to evidence-based treatment. One solution to address these inequities is a team-based care (TBC) model, defined as one in which at least two providers work collaboratively with a patient and their caregiver(s) to make healthcare decisions. This paper seeks to describe the implementation of a TBC model within a safety-net healthcare setting and determine the extent to which it can be an effective, patient-centered approach to treating individuals with diabetes.

**Methods:**

Semi-structured interviews were conducted with staff (*n* = 15) and patients (*n* = 18). Clinical data were extracted from the electronic medical record of patients (*n* = 1,599) seen at a safety-net health system in Chicago, Illinois, United States. The mixed methods study was guided by implementation science and participatory research principles. Staff interviews were 60 min and covered patient care activities, work flow, perceived patient experience, and facilitators/barriers to care coordination. Patient interviews were 60 min and covered satisfaction, attitudes about diabetes management, quality of life, and technology. Patient interviews were co-analyzed by research staff and members of a patient advisory committee. Clinical data were collected at an index visit, two years prior and at one-year follow up (*n* = 1,599).

**Results:**

Four themes emerged from the interviews: (1) patients perceived the TBC model to be patient centered and of high quality; (2) technology can be an innovative tool, but barriers exist; (3) diabetes management is a complex process; and (4) staff communication enhances care coordination, but misinterpreting roles reduces care coordination. From pre-enrollment to the follow-up period, we found a statistically significant increase in missed visits, decrease in hemoglobin A1c (HbA1c), decrease in body mass index, and decrease in the percent of patients with high blood pressure. We found that each medical visit during the follow-up period was associated with an HbA1c decrease of 0.26 points.

**Conclusions:**

A TBC model is a patient-centered approach to providing care to patients with complex health needs, such as diabetes, patients were satisfied with the care they were receiving, and the model was associated with an improvement in clinical outcomes.

**Supplementary Information:**

The online version contains supplementary material available at 10.1186/s12913-024-11062-4.

## Background

In 2021, the United States (U.S.) Centers for Disease Control and Prevention (CDC) reported 38.1 million U.S. adults aged 18 years or older—or 14.7% of all U.S. adults—were living with diabetes [1]. It is estimated that an additional 8.5 million U.S. adults have undiagnosed diabetes, demonstrating the burden of disease that requires coordinated care and quality treatment [2]. In addition to high prevalence, the total cost of diagnosed diabetes continues to rise. Between 2012 and 2017, the total cost increased by 26% [3]. Like other chronic diseases, the burden of diabetes management lies heavily upon the patient and requires strict medication adherence, regular monitoring of glucose levels, intentional eating habits and other behavioral interventions [4].

Individuals facing socioeconomic hardship experience higher than average rates of chronic disease, with less access to evidence-based treatment. Research has shown that low-income patients have higher chronic disease prevalence rates, incidence rates, and per patient disease-related costs compared to the national estimates for all adults [5–7]. Data from the 2021 Illinois Diabetes Burden report showed a significantly higher diabetes prevalence for those with an annual household income less than $15,000 [8].

One solution to address the needs of patients with complex health conditions is a team-based care (TBC) model. TBC is defined by the Institute of Medicine as “the provision of health services to individuals, families, and/or their communities by at least two health providers who work collaboratively with patients and their caregivers—to the extent preferred by each patient to accomplish shared goals within and across settings to achieve coordinated, high-quality care” [9]. Existing literature suggests that TBC can be effective in managing diabetes, specifically in lowering HbA1c, blood pressure, and cholesterol [10]. The Community Preventive Services Task Force also found TBC to be patient-centered and flexible in a variety of settings. However, less is known about its effectiveness as a model of care within healthcare settings primarily serving racial and ethnic minorities and/or low-income patients [11–16]. The present study seeks to fill a gap in the current literature around the implementation of a TBC model with a multi-disciplinary team in a safety-net health system that serves predominately low-income patients of color. The purpose of this paper is to describe the implementation of a TBC model for patients living with diabetes who receive care at a safety-net health system, describe patient satisfaction of the model, and measure changes to utilization of health care services and diabetes-related health outcomes.

## Methods

### Setting

In Chicago, Illinois, 12.4% of adults are living with diabetes [17]. Like other parts of the U.S., people of color (POC) experience disproportionately higher rates compared to their white counterparts. Nearly 17% of non-Hispanic Black individuals in Chicago are living with diabetes compared to 12.3% of Hispanic individuals, and just 8.9% of white individuals [17]. Rates of diabetes are also unequally distributed across geographic regions. Those on the West and Southwest sides of the city, communities which are predominately comprised of POC, have rates as high as 33.2%. Yet the highest neighborhood-level prevalence rate on the North side, predominantly comprised of white individuals, is 18.6% [17].

Sinai Chicago is the largest private safety-net health system in Illinois. In the U.S., a safety-net health system is a designation for systems that are obligated to provide care for all individuals, regardless of their insurance status or ability to pay. This often results in high proportions of uninsured or underinsured and low-income patients. Sinai Chicago serves the historically disinvested communities on the West and Southwest sides of Chicago, which are comprised of predominately un- or under-insured POC [18]. Many of Sinai’s patients also experience a disproportionate burden of chronic diseases such as diabetes, cancer, and heart disease. In many communities served by Sinai Chicago, an estimated 30–50% of residents have a family history of diabetes [19].

### Team-based care model

For decades, Sinai primary care providers, endocrinologists, nurses, and dieticians have provided clinical care and individualized diabetes self-management education to patients with complex clinical and social needs. The patient would see separate providers, on different days, who may review the medical record, but not engage with other providers in a manner to collectively address patient goals. However, this model lacked the integration that is required for adequate and holistic management of uncontrolled diabetes often seen in this population. In response, Sinai launched the Center for Diabetes and Endocrinology (the Center) in September 2020 with the goal of establishing a destination of choice for patients with diabetes in Sinai’s primary service area. The Center treats patients with prediabetes, diabetes (types 1 and 2 and gestational), and other endocrine disorders in a multidisciplinary setting that offers education, nutrition, and prevention. This robust, patient-centered program provides streamlined services to address the full spectrum of patients’ medical (physical, pharmaceutical, and behavioral) and non-medical (nutrition, social, and emotional) needs in one clinical setting. The Center’s clinical team addresses physical needs from mild to severe cases and is supported by pharmacy and a variety of wraparound services (social work, community health workers). Throughout the patient experience, technology is integrated to ensure ongoing monitoring of patient adherence to treatment plans, rapid response to changes in disease status, and educational outreach. For example, patients received access to telehealth appointments to encourage appointment adherence. Similarly, patients may be offered remote glucose monitoring (RGM) which allows for blood glucose data to be integrated into the medical record and shared between the patient and provider for proactive follow up. Medical assistants, pharmacists and diabetes educators review blood glucose data and contact patients who show clinically concerning changes in glucose levels.

### Study design and frameworks

We conducted a convergent mixed methods process and outcome evaluation guided by implementation science and participatory research principles [20]. The objectives of this study were to: (1) describe the implementation of a TBC model for patients living with diabetes who receive care at a safety-net health system; (2) describe patient satisfaction and the extent to which the Center engages patients and responds to their needs; and (3) measure changes to utilization of health care services and diabetes-related health outcomes.

**Fig. 1:**
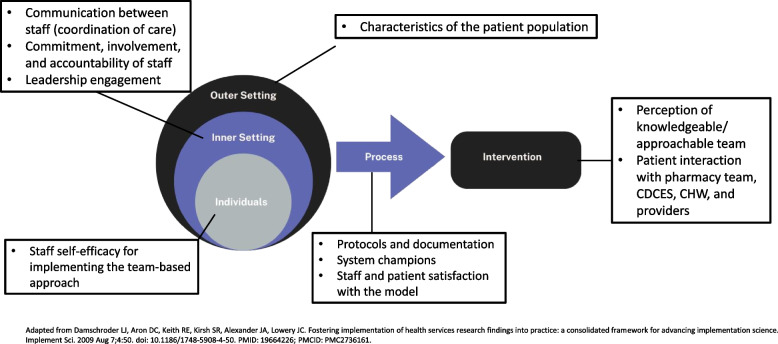
Conceptual framework for evaluating the Sinai center for diabetes and endocrinology

We employed the Consolidated Framework for Implementation Research (CFIR) to guide our understanding of the various components of the Center’s TBC model [21]. CFIR is one of the most popular frameworks for implementation research and can be used to identify the factors that may influence effectiveness of the implementation process [22]. This framework is organized into five domains: the intervention, the inner setting, the outer setting, the individuals involved in the intervention, and the process of implementation. Within the context of this study, the outer setting refers to Sinai Chicago’s patient population, the inner setting refers to the Center, the individuals refer to both the providers implementing the TBC model and the patients receiving care, the process refers to the implementation of the intervention (TBC), and the intervention refers to the TBC model. The research team identified implementation constructs for each domain to guide the overall evaluation (Fig. 1).

The evaluation also employed a community-based participatory (CBP) approach by including community residents with lived experience in our data collection, analysis, and interpretation processes [23]. At the project onset, the research team, with extensive experience in community-engaged research, convened a Patient Advisory Committee (PAC) [24]. The PAC comprised seven individuals 18 years of age or older who are living with diabetes. The PAC met almost every other month for 18 months. PAC meetings were organized and led by the research team. Topics included: development of the patient interview guide, human subjects research training, qualitative data analysis training, coding and theme development, sense-making, and developing dissemination products. We followed CBP research principles by building on strengths within the community, recognizing and highlighting the lived experience of individuals living with diabetes, facilitating partnership between community residents and researchers, and encouraging co-learning and empowerment [25]. The Mount Sinai Hospital (MSH) Institutional Review Board approved this project (protocol #21–37).

### Data collection and analysis

The study team conducted semi-structured interviews with Center staff and patients, and extracted clinical data from the electronic medical record (EMR). Data from our qualitative and quantitative methods were collected and analyzed separately, and findings were merged to achieve study objects.

#### Semi-structured interviews

Sixty-minute semi-structured interviews were conducted with 15 Center staff, who were recruited through email. Any staff employed by the Center were eligible to participate. Interview guides were created to align with the relevant implementation domain and constructs from Fig. 1. For example, interviews with staff included questions related to: the inner setting (communication between staff, involvement and accountability of staff, and leadership engagement), the intervention (daily activities, the degree to which the Center is appropriate for the target population), the individuals (perceived patient experiences), and the process (work flows and protocols, facilitators and barriers to coordination across the Center). The full staff interview guide can be found in the supplemental materials.

Sixty-minute semi-structured interviews were also conducted with 18 patients. Patients were recruited via Sinai’s social media platforms, flyers, and referrals from Center staff. The research team trained two community health workers (CHW) to conduct all patient interviews. The CHWs who served as interviewers are from the target communities, but not affiliated with the Center. Interviews were conducted in English or Spanish. Patients were eligible to participate if they were 18 years of age or older, an active patient of the Center (had at least one appointment during the study period), currently living with pre-diabetes, diabetes or other endocrine disorder and competent to provide consent. The interview guide was developed to align with the relevant implementation domain and constructs from Fig. 1 and in collaboration with the PAC to ensure that members of the community informed the research process. The interview guide included questions related to: process (satisfaction with their care at the Center), intervention (knowledge, attitudes and beliefs about diabetes management; quality of life; perceptions of the care team; experience with education received from staff; perceptions of how the Center influences health), individuals (attitudes and experiences with patient-centered technology, application of diabetes education). The full patient interview guide can be found in the supplemental materials.

All interviews were conducted virtually using a HIPAA-compliant version of Webex, and audio recorded with permission. Recordings were transcribed verbatim by a professional transcription company. Following the qualitative analysis training for PAC members, the research team led two virtual coding sessions with the PAC to develop initial codes for a subset of transcripts. The research team incorporated the feedback from the PAC into the final codebook.

Transcribed interviews were coded using QSR NVivo. Two researchers coded the English interviews and two bilingual (English and Spanish) researchers coded the Spanish interviews following the usual standards of qualitative research analysis [26, 27]. The researchers followed a grounded theory approach to analyze interview data [28]. Each reviewed the transcriptions to determine themes and sub-themes, reviewed each other’s themes and discussed discrepancies to arrive at consensus. The findings were then narrowed based on the most significant themes. After conducting the initial analysis, the research team presented themes and sub-themes back to the PAC in sense-making sessions to understand results, elicit contextual feedback and ensure that interpretations accurately reflect the insights of patients.

#### Patient clinical data

Outcomes of the TBC model and patient’s health outcomes were assessed via EMR data. Each patient’s index visit was defined as their first visit after the formation of the Center. Up to three years of data were collected for each patient: two years prior to the index visit, and one year of follow-up after the index visit.

##### Outcome variables

Clinical outcomes of interest were HbA1c, body mass index (BMI), and elevated blood pressure. HbA1c and BMI were treated as continuous variables. Blood pressure was dichotomized into elevated blood pressure (systolic blood pressure [SBP] ≥ 130 mmHg or diastolic blood pressure [DBP] ≥ 80 mmHg) and not elevated blood pressure (SBP < 130 mmHg and DBP < 80 mmHg) [29]. Healthcare utilization outcomes of interest were the number and type of visits completed (e.g., endocrinology-related medical visits, community health worker visits, other visits such as retinal eye exam and diabetes educator visits), the number of missed visits, emergency department visits, and inpatient hospitalizations. The type of visit was defined by categorizing the text typed into the “event” and “location” fields of each visit record in the dataset. Additionally, a binary healthcare utilization variable was created using emergency department visits and inpatient hospitalizations (zero emergency department visits or inpatient hospitalizations vs. at least one emergency department or inpatient hospitalization).

##### Explanatory variables and covariates

Comorbidities were categorized into three groups: concordant microvascular (including renal disease, neuropathy, and eye disease), concordant macrovascular (coronary disease, cerebrovascular disease, hypertension, and heart failure), and discordant (arthritis, cancer, and chronic lower respiratory disease) [30]. Sociodemographic variables included age, race/ethnicity, language, and insurance type. Age was treated as a continuous variable; race/ethnicity was categorized into non-Hispanic Black, non-Hispanic White, Hispanic/Latino, and Other/Unknown; language was categorized into English, Spanish and Other/Unknown, and insurance type was dichotomized into private insurance and other coverage (Medicaid, Medicare, charity, other/unknown).

##### Analysis

Patient health outcomes at the index visit were compared to those one-year post-enrollment. We considered the data closest to the date of the index visit (within 60 days) to be the index measurement. The last measurement collected before the end of the 12-month follow-up period was considered the post-enrollment measurement. The association between index and post-enrollment blood pressure outcomes were tested for significance using a χ^2^ square test; all other health outcomes were tested using paired t-tests.

The annual rate of missed visits, emergency department visits, and inpatient hospitalizations during the pre-enrollment period was compared to the follow-up period. The annual pre-enrollment rate was calculated as the average rate over the two-year pre-enrollment data collection period. Paired t-tests were used to compare pre- and post-enrollment rates.

Finally, linear and logistic regression models were fit to determine which variables, if any, were significantly associated with patient health- and health care utilization outcomes. Regression models were developed using a step-wise reduction approach. A *p*-value < 0.05 was considered statistically significant. Quantitative analyses were conducted using Stata version 15.1 (StataCorp LP, College Station, TX).

## Results

Data from semi-structured interviews with staff and patients and electronic medical record data on health care service utilization were triangulated to provide insights into the implementation of the TBC model and utilization of health care services at the Center. Fifteen Center Staff and 18 patients were interviewed. Participating staff roles included: CHW, certified diabetes care and education specialist (CDCES), pharmacist, endocrinologist, registered nurse, nurse practitioner, medical assistant, and administrator. The majority of patients interviewed were female (56%), Hispanic or Latinx (69%), over 50 years of age (75%), had less than a high school diploma (56%), and living with type 2 diabetes (50%). Clinical data was analyzed for 1,599 Center patients. Two-thirds of those patients were female, the mean age was 51.5 years, and the majority (69%) were English-speaking. Among known race/ethnicity, 54% were Hispanic/Latinx and 40% were Non-Hispanic Black. Nearly half (45%) of patients were on Medicaid, 44% of patients had one or more concordant microvascular comorbidity, 55% had concordant macrovascular comorbidity, and 26% had discordant comorbidity (Table 1). The following describes results from the triangulation of qualitative and quantitative analyses. First, we will describe the implementation of the TBC model in practice, including routine care metrics and patient satisfaction, and then we will describe how health care service utilization and health outcomes changed over time Table.

**Table 1:**
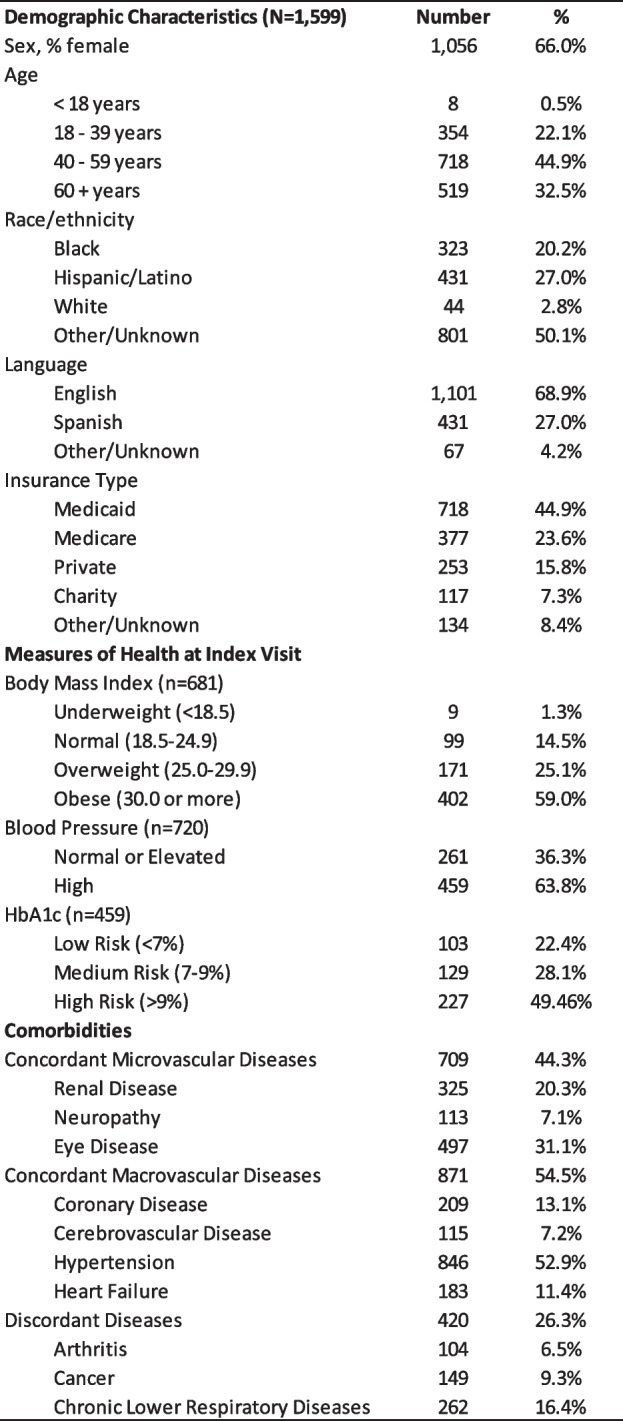
Demographic & Health Characteristics of Patients enrolled in the Center between August 2020 to June 2021 (*n* = 1,599)

### Implementation of a TBC model and patient satisfaction

Based on the analyses of interview data collected from patients and staff, three themes emerged related to TBC implementation and patient satisfaction: (1) patients perceived the TBC model to be patient centered and of high quality; (2) technology has the potential to be an innovative tool; and (3) staff communication can enhance care coordination. Additional illustrative quotes are provided in Table 2.

**Table 2:**
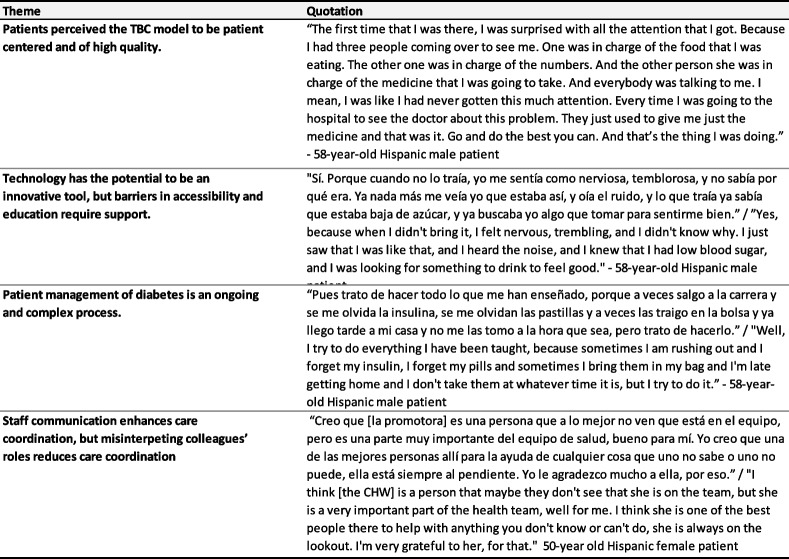
Themes and illustrative quotations

#### Patients perceived the TBC model to be patient centered and of high quality

Patients interviewed described high levels of satisfaction with the TBC model employed by the Center. Patients consistently mentioned feeling important and cared for from their very first appointment at the Center and thereafter. As one patient shared:

*“I mean when my first visit in – I saw three people talking to me about the things that they can do for me. And they make me feel like I’m really important… I mean, they made me feel part of the team. They made me feel like I’m really important to them. And that’s – I mean, I have never felt this before with nobody.*” 58-year-old Hispanic patient

Patients reported that having access to a variety of services in one place felt coordinated, and supported patients’ diverse needs. There was a wide range of the number of visits patients had with each provider type, with a mean of 2.81 visits, 0.61 visits, and 3.88 visits with an endocrinologist, community health worker (CHW), or other provider (e.g., pharmacist, diabetes education or dietitian), respectively. During interviews, several patients mentioned Center staff by name and felt that coordination between staff ensured patients had an advocate with insurance companies, access to medication or medical equipment, support to improve diet and nutrition, and were provided a bilingual staff as needed. Specifically, Spanish-speaking patients were grateful to have access to bilingual staff, including a CHW, who could provide clarity on providers’ treatment instructions, help patients understand and navigate health insurance and the cost of healthcare for uninsured individuals, and offer diabetes health education in Spanish.

*“La ayuda que siempre me ha brindado [la promotora], ella siempre está atenta, pendiente de ayudarlo a uno, ella me ha ayudado muchísimo, lo que yo no entiendo, lo que yo no puedo hacer, ella hasta me ha ayudado a ir donde uno va para los pagos, se me olvido el nombre.” / “The help that [the CHW] has always given me, she is always attentive, always trying to help me, she has helped me a lot, what I do not understand, what I cannot do, she has even helped me to go where I go for payments.”* 50-year-old Hispanic patient

Patients believed the multi-disciplinary TBC model was comprehensive, thorough, and centered around their needs.

#### Technology has the potential to be an innovative tool

The Center considers the use of technology to be an innovative tool to support patient’s diabetes self-management. For example, continuous glucose monitoring (CGM) is an automated process of tracking blood sugar levels throughout the day using a CGM device. Remote glucose monitoring (RGM) refers to the process of transmitting that information directly from a personal smartphone device to a healthcare provider using a remote data transmitter. Using a mobile application, patients can: document their glucose levels; sync data from a meter, insulin pump, pen or continuous glucose monitoring device; track their weight; log food and meals; access educational resources; track steps; and track blood pressure. Remotely uploading data provides patients with a direct line to their provider for regular feedback on progress and answers to questions between clinic visits. As one staff member describes:

*If patients are able to be set up with a continuous glucose monitor they really like it, because it's giving them a lot more data about their blood sugars. Tools like that could be life changing for a patient.* Diabetes Center Staff*,* Registered Nurse

Patients reported feeling more connected to their care and considered this to be a patient-centered tool.

*I know what my sugars are now. And that I didn’t know for a long time. They got me a machine where I know my sugar at all times.* 50-year-old Black patient

Yet patients also expressed barriers to utilizing technology. Some patients expressed a lack of knowledge about CGM/RGM, or frustrations when it broke. There was a desire to use technology, however patients need high-touch and regular support on how to use the devices, and use the data from the devices. As one patient describes,

*“Lo que pasa es que yo no sé si perdí una cita o no me la dieron y ya dejé de ponerme este aparatito porque es para 10 días nada más, ya después ya no me sirve.” /* “What happened is that I don't know if I missed an appointment or they didn't give it to me and I stopped using this little device because it is only for 10 days, after that it stopped working for me.” 58-year-old Hispanic patient

#### Communication enhances care coordination

Center staff reported that information sharing across roles was underpinned by regular and varied modes of communication. Staff indicated that coordination across staff may happen through email, EMR documentation, team meetings, or curbside consultation, where information is shared informally and verbally.

*All through EHR [*Electronic Health Record*], because they all document as they go. So when the MA* [Medical Assistant] *sees them and takes the vitals, she documents everything on EHR and then the endocrinologist does the same thing and then it just goes down. And then as they're rotating in and out, they talk to each other to let each other know what's going on.* Diabetes Center Staff*,* Receptionist

Yet, care coordination was threatened by the lack of knowledge each staff person had for other roles. For example, one staff member had a dual role as a CHW and a retinal specialist. Several staff were aware of his work as a retinal specialist, but were not aware he was a CHW and what services he could provide. This ultimately impacted their ability to deliver the full range of services available within the Center. Further, staff reported different levels of team engagement to discuss cases. When asked if the healthcare team ever meet to make decisions about a patient’s care, an endocrinology stated “No, not mutually. At least not with my patients. I don’t know what anyone else does.” However, when this was asked of a nurse, she replied “Either before or while the patients there. Yeah, if needed we would all coordinate about what could help the patient [with]… the nurse, the doctor, and the pharmacy team.”

### Urgent care and clinical outcomes

A fourth theme that emerged from the data is that despite the TBC model and wraparound support, diabetes management is complicated and an ongoing process. Patients discussed the complexities of managing diabetes despite the TBC model and in some cases, poor control was attributed to their lack of self-management. In other cases, patients expressed awareness that despite their adherence to treatment regimens recommended by their clinician, they still did not feel they were able to maintain control. Patients reflected on the challenges of managing stress, maintaining healthy eating habits, and getting enough rest. As one patient shared:

*Well, my latest appointment with the nurse, I didn’t do my best for this time around, getting checked. My A1c did go up just a little bit only because it’s been a stressful time for me. My grandma passed away recently, so that was hard for me, and things just got difficult. I started not paying attention to what I was eating.* 30-year-old Hispanic patient

Patients reflected on their experience living with diabetes and acknowledged the resources that it takes to properly manage their disease. They identified the need to have a good support system, connecting with others who have “healthy” habits, and even managing challenges.

The challenge in diabetes management is strengthened by the analysis of clinical data. Index data was collected from August 2020 to June 2021, pre-enrollment data went back as far as August 2018, and follow-up data was collected through June 2022. In the two years prior to the Center, 9% of patients had at least one emergency department visit at Mt. Sinai Hospital (mean of 0.07 visits per patient) and 5% were hospitalized at least once at Mt. Sinai Hospital (mean of 0.04 hospitalizations per patient). These percentages were not statistically significantly different at the follow-up period (4% and 2%, respectively). The mean number of missed visits significantly increased from 4.71 at pre-enrollment to 8.54 in the follow-up period (*p* < 0.001). There was a statistically significant decrease in HbA1c from the index to the follow-up period (9.28% to 8.08%, *p* < 0.001). Small but statistically significant changes were also seen in BMI (33.16 kg/m^2^ to 32.79 kg/m^2^, *p* = 0.01) and the percent of patients with elevated blood pressure (64.39% to 60.43%, *p* < 0.001) (Tables 3 and 4).

**Table 3:** Measures of the team-based care model implementation and key patient outcome

**a. Mean visits and range by visit type after team-based care implementation**			
Routine Care (# of visits post-enrollment)	Mean	Range	
Endocrinology-related medical visits	2.81	0-19	
CHW visits	0.61	0-10	
Other visits	3.88	0-44	
**b. Health care utilization and clinical outcomes pre, during, and post-team based care implementation**			
	Pre-TBC	TBC Follow-up	p-value
Missed Visits, annually (#)	4.71	8.54	<.001
Urgent Care, annually (#)			
Emergency Department visits	0.07	0.06	0.36
Inpatient hospitalizations	0.04	0.04	0.63
Health Outcomes	Index Visit	TBC Follow-up	p-value
HbA1c level, n=296 (%)	9.28	8.08	<.001
Weight, n=534 (lbs)	201.05	198.98	0.007
Body Mass Index, n=501 (kg/m^2^)	33.16	32.79	0.01
Elevated Blood Pressure, n=556 (%)	64.39%	60.43%	<.001

Paired t-test were used to assess statistical testing except for % high blood pressure, where a Chi Square test was used. Other visits included those with a diabetes educator, dietician, pharmacist

*TBC* Team-based care

**Table 4:**
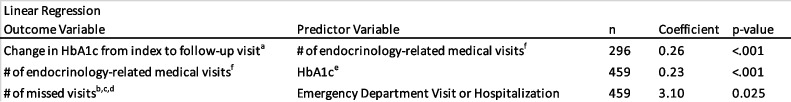
Multiple linear regression coefficients and multiple logistic regression odds ratios for associated predictor and outcome variable

Adjusted for: ^a^Sex, ^b^Concordant microvascular comorbidities (renal disease, neuropathy, and/or eye disease), ^c^Discordant comorbidities (arthritis, cancer, and/or chronic lower respiratory disease), ^d^Age. Time period: ^e^Index Visit; ^f^Follow-up Visit

The regression analysis focused on four outcome variables: change in HbA1c from index to post-enrollment, the number of endocrinology-related medical visits, number of missed visits, and percent of patients with at least one instance of healthcare utilization. After controlling for sex, we found that each provider visit during the follow-up period was associated with an HbA1c decrease of 0.26 points from index to post-enrollment (*p* < 0.001). Urgent health care utilization during the follow-up period was associated with an additional 3.1 missed visits during the follow-up period compared to patients with no urgent health care utilization, after controlling for concordant microvascular comorbidities, discordant comorbidities, and HbA1c at the index visit (*p* < 0.001). In a logistic regression model, we found a 26% increase in the odds of a hospitalization or emergency department visit for every 1.0% increase in HbA1c at the index visit compared to their pre-enrollment HbA1c, after controlling for concordant microvascular comorbidities and age (*p* < 0.001) (see Table 4).

## Discussion

Diabetes is the seventh leading cause of death in the United States, and is associated with heart disease, vision loss, kidney disease, and even death [31]. Evidence-based interventions and tailored approaches to implementation are critical to appropriately address the high rates of diabetes and prediabetes, and equitably provide care to patients. The present study offers important findings from a process and outcome evaluation of a multi-disciplinary TBC model for patients living with diabetes. This is particularly important for low-income and racially diverse patients with co-occurring comorbidities who, like other patients served by health systems in the U.S. safety net, often have more complex needs that require the additional social support and wraparound services provided at the Center. For instance, these patients are more likely to have competing priorities or child-care or transportation needs that prevent them from attending multiple appointments on different days [32].

The triangulation of key findings from staff and patient interviews, and clinical data describe the implementation of the TBC model at the Center and illuminate the components of the model that satisfy patient needs and preferences, facilitate coordination of care, and even components that require adjustments in protocol or approach. Patients expressed high levels of satisfaction with the quality of care they received, noting attentive staff and noticeable coordination among clinical staff regarding their care. This is aligned with other research of TBC models, in which slight increases in patient satisfaction were documented [33].

Some research suggests that patients who regularly use CGM/RGM technology have better clinical outcomes than patients who are not actively engaged [34]. Our findings support the perceived benefits of CGM/RGM, but also identified barriers during real-world implementation that must be addressed such as insurance coverage, knowledge around utilization, resources for troubleshooting, and access to smart phones for ideal integration. We found that with high-touch support from staff, these barriers can be overcome.

We hypothesized that by increasing the availability of telehealth appointments, prompted by the emergence of the COVID-19 pandemic, along with the TBC model, we would see a decrease in missed appointments. Counter to this hypothesis, missed appointments increased from pre to post TBC implementation. Existing research of TBC models have not explored the impact of this model on missed visits. The Center and its TBC model were launched at the onset of the COVID-19 pandemic which may have necessitated patients’ prioritization of other more urgent issues such as childcare, food accessibility and other essential daily needs. It’s also possible that during the transition, there was confusion about how to schedule appointments with new providers (i.e., pharmacists, CHWs) or where to go to meet them. However, we also hypothesized that uncontrolled A1c at the index visit would lead to an increased likelihood in attended endocrinology-related visits, which was confirmed. This may be because patients had newfound access to additional resources within the clinic (i.e., appointments with a diabetes educator, pharmacist, etc.).

The TBC model evaluated in the present study included several non-clinical staff members such as CDCES and CHWs. Multi-disciplinary teams that include both clinical and non-clinical staff have been shown to improve patient outcomes and improve social determinants of health [35]. Interventions with CHWs as non-clinical staff, specifically, have been recommended based on improved outcomes in diabetes and cardiovascular management, among other areas [36]. CHWs integrated within traditional clinical care teams can address the complex health-related social needs within the individual and community context of the patients, which we found can serve as barriers to proper diabetes self-management, and are not typically within the scope of clinical providers. As of July 2022, 29 US states reported that they allow payments through Medicaid for CHW-related services, but further work is needed to effectively integrate, supervise, and sustain CHW efforts within the healthcare system and community settings at large [37].

Implementation science frameworks, such as CFIR, provide a guide for how to measure, and even improve, the integration of promising interventions into routine practice [38]. The complexities of systemic racism, organizational infrastructure, and unique patient needs require public health professionals and clinicians to think differently about impactful interventions for patients and the strategies used to implement these interventions. Our study used the CFIR framework to systematically assess and understand multilevel factors that may influence the implementation of, and outcomes related to a TBC model. To strengthen our methodology, we paired the implementation science approach with participatory methods. Studies that include stakeholders in the research process, often have high-quality processes and outcomes, developed capacity and competency of stakeholders, and higher likelihood of sustainability [39, 40].

While our study provides evidence for how TBC can impact patient outcomes, we did not examine TBC sustainability or maintenance, particularly as it relates to cost effectiveness. The US traditionally uses a fee-for-service payment model, but this model been criticized for incentivizing providers to perform more procedures rather than focus on outcomes. Other payment models have been tested such as accountable care organizations, bundled payments, and patient-centered medical homes. A systematic review from 2023 found that TBC for blood pressure control was cost effective and had a significant impact on the quality adjusted life years of patients. However, this review also found that compared to other high-income countries, the US intervention costs were higher and varied by composition of the care team and racial/ethnic makeup of the patient population. Future research should examine the degree to which a TBC model for low-income communities of color living with diabetes has the potential for long-term cost savings.

There are several strengths to this study. First, our partnership with the PAC ensured that our data collection tools, recruitment approaches, analyses, and interpretation were guided by lived experience. Their meaningful participation improved the quality of the study. Second, because the Center was opened within an existing patient population, this presented a unique opportunity to capture data on the same patients at three different time points: before the opening of the Center, at an index visit, and at a one-year follow up. Third, our triangulation of qualitative and quantitative data allows us to more fully understand the impact of the TBC model on Sinai’s patient population.

There are also important limitations that must be noted. First, emergency department and hospitalization data was only available for Mt. Sinai Hospital. If patients visited an emergency department or were admitted at another hospital, this data was not included in our analysis. Second, nearly half of race/ethnicity data was missing from our EMR data. Third, it was not possible to easily identify unique patient meetings with a pharmacist, diabetes educator, or retinal specialist. These meetings were only documented as a qualitative note in the medical record and could not be easily extracted. It was also not possible to isolate endocrine-related visits across the same study population prior to the implementation of the TBC model. Fourth, this study began just six months after the U.S. declared COVID-19 to be a public health emergency, as a result, patient volume was initially lower than expected and took several months to increase. Finally, because this was an observational study, we are not able to conclude these findings are solely attributed to the effects of the TBC model. They may be due to unmeasured factors such as implementation of telemedicine, the ongoing effects of the COVID-19 pandemic, changes in staff, etc.

## Conclusions

In summary, a multi-disciplinary TBC model can be an appropriate and effective model to improve health outcomes for patients living with diabetes in an urban setting with complex needs. Patients perceived this to be a patient-centered model, and improved patient outcomes were documented compared to prior to the implementation of this model. Specifically, we found a reduction in HbA1, weight, BMI, and proportion of patients with elevated blood pressure. Improvements can be made by ensuring clear understanding of staff roles. These findings suggest that despite integrating clinical and social service providers in one space, a TBC model is not a panacea for addressing complex chronic disease and social needs for patients from low-income communities. Policy changes such as expanded health insurance, adjustments to the healthcare payment system, and additional funding for national and local efforts to address social determinants of health are required for broad change. As of the writing of this manuscript, the TBC model of care is still being offered at Sinai Chicago, and research continues using advanced study designs to measure causal effects.

### Supplementary Information

Supplementary Material 1. 

## Data Availability

The datasets used and analyzed during the current study are available from the corresponding author on reasonable request.

## Abbreviations

US: United States
CDC: Centers for Disease Control and Prevention
TBC: Team-based care
POC: People of color
CFIR: Consolidated Framework for Implementation Research
CBR: Community-based participatory
MSH: Mount Sinai Hospital
PAC: Patient Advisory Committee
EMR: Electronic medical record
CHW: Community health worker
BMI: Body mass index
SBP: Systolic blood pressure
DBP: Diastolic blood pressure
DCES: Diabetes care and education specialist
CGM: Continuous glucose monitoring
RGM: Remote glucose monitoring
HbA1c: Hemoglobin A1c
