# Supplementary material for: Impact of a multi-disciplinary team-based care model for patients living with diabetes on health outcomes: a mixed-methods study
Source: BMC Health Serv Res. 2024 Jun 18;24:746. doi: 10.1186/s12913-024-11062-4 (PMC11186232; doi:10.1186/s12913-024-11062-4)
Supplement: Supplementary file 1 — Supplementary Material 1. [file 12913_2024_11062_MOESM1_ESM.docx]

# Sinai Center for Diabetes and Endocrinology Staff Interview Guide

***Intro and Consent Process (5 min)***

<<Interviewers introduce yourselves and read the following>> Thank you for meeting with us to discuss your work at the Center. Before we start the interview, I want to make sure that you understand what is going to happen and confirm that you’d like to participate.

<<Read verbal consent script to participant>>

Now we are ready to get started. I’m going to start the recorder and <<note taker’s name>> will be taking some notes while we talk. We are looking for your honest feedback so we can learn what factors impact patient outcomes, so don’t be afraid to share your thoughts.

***General: Daily Activities/Workflow*** ***(15 min)***

1. What is your role at the Center?
2. What does a typical day look like?

Probe: Is there anything else you have been involved in, even if it’s not a regular activity?

1. Let’s talk a little bit about the patient experience. Can you start by walking us through what happens when a new patient contacts the Center to schedule their first appointment? And then what happens (all the way through an appointment, follow-ups, etc)?

Probe: Make sure to ask their perception of if/when a patient sees a CHW, CDCES, pharmacist, clinician, social worker

Probe: How is this experience different if the patient is an existing patient?

Probe: What kinds of appointments might a patient schedule?

1. What kinds of tools do patients receive to assist in improving their health outcomes?

Probe: nutritional counseling, technology to monitor blood glucose levels

Probe: In general, do you feel like these tools are well received?

***Coordination of Care: Facilitators and Barriers (20 min)***

1. You mentioned that in your role you typically do <<refer to Q2, Q3>>. How are these activities that you perform with a patient shared with others on the care team? Probe: How do others on the care team know if a patient received nutritional counseling, referrals, wrap-around services?
2. Similar to the last question, how do you get information about the patient’s care from others on the care team?

Probe: curbside consultations? Documented in the EMR? Where in the EMR?

1. How often does the healthcare team meet to make decisions about each patient’s care plan?

Probe: Who do you consider to be part of the healthcare team?

1. Think back to the last time you met with the healthcare team.

Who was there (either name the individual or their position)?

What was that meeting like and what was the purpose?

What was the goal of that meeting?

What did each person contribute and how do you feel like their expertise helped to determine the patient’s care plan?

1. What’s the process for sharing information when transferring care for a patient?
2. We are particularly interested in how this model of care provides support to patients across a wide range of clinical and social needs. Have you ever identified a patient need that could not be addressed by the healthcare team? If so, how have you addressed this?

Probe: If you have partners in the community you refer to, who are they?

1. What do you think helps coordinate patient care?
2. What has made it difficult to coordinate patient care?
3. [For leadership role]: Can you tell us a little bit about the Center’s relationship with the managed care organizations and Medical Home Network?

Probe: How are priorities aligned?

***Perceived Patient Perceptions (15 min)***

1. How are patients engaged in developing their plan of care?
2. How do patients know who their primary contact is on the care team?
3. What is your perception of patient satisfaction with this model of care?

***Wrap-up (5 min)***

1. Is there anything else you’d like to share that we didn’t touch on today?

<<Thank participant.>>

# Sinai Center for Diabetes and Endocrinology Patient Interview Guide

***Intro and Consent Process (5 min)***

<<Interviewers introduce yourselves and read the following>> Thank you for meeting with us to discuss your experience as a patient at Sinai’s Center for Diabetes and Endocrinology. As you may know, Sinai Chicago opened the Center for Diabetes and Endocrinology in September of 2020. The goal of this Center is to become a destination of choice for patients with diabetes in the communities served by Sinai. The Center uses a team approach to treat patients with prediabetes, Types 1 and 2 diabetes, gestational diabetes and other endocrine disorders. We are speaking to a number of Center patients, like yourself, to get a sense of the perspective of diabetes in your community, the Center, and how to best promote and build models of care that are culturally competent for Sinai’s patients living with diabetes. Before we start the interview, I want to make sure that you understand what is going to happen and confirm that you’d like to participate.

<<Read verbal consent script to participant>>

Now we are ready to get started. I’m going to start the recorder << if there is a note taker, introduce them and mention they will be taking notes during the interview>>. We are looking for your honest feedback so we can learn how you benefit from the Center and what you find challenging, so please don’t be afraid to share your thoughts. Any questions before I get started?

***General: Patient Overview (10 min)***

1. How long have you been living with diabetes (or pre-diabetes)?
2. How long have you been receiving care for your diabetes/prediabetes at Sinai?

Probe: Have your appointments been in person? Telehealth? Both?

1. Were you a Sinai patient before the Center was established?

Probe: If you have been a patient at Sinai for over a year, how has your experience with your care changed since the Covid-19 pandemic?

Probe: If you were diabetes diagnosis was at another medical group or hospital, how has your experience at the Center/Sinai been different?

1. Why have you decided to receive care at Sinai as opposed to another health system?

Probe: Did you receive a referral before visiting the Center/scheduling a telehealth appointment?

Probe: Is this decision based on insurance coverage?

Probe: Is it geographically convenient for you?

Probe: Is there something about the quality of care or the model of care that is attractive to you?

***Center Patient Experience (20 min)***

**5a. **Ask if the patient started receiving care at Sinai since September 2020 or later.****

Let’s talk a little bit about your first experience at the Center back in <<Month of First Appointment>>. Can you walk us through that first appointment, and what that was like?

Probe: How did you make an appointment?

Probe: Was this appointment in person or via telehealth?

Probe: How was your experience finding the center if you went in person?

Probe: Who did you see/meet with? Endocrinologist, PCP, CHW, CDCES, pharmacist, clinician, social worker? If so, what did you think of those visits?

Probe: What kinds of appointments have you scheduled since then?

**5b. **Ask if they have been an existing Sinai patient since before September 2020****

As described earlier, the Sinai Center for Diabetes shifted their model of care in September 2020 to be more comprehensive and include appointments with an endocrinologist, nurse educator, pharmacist, and retinal tech in the same visit. Depending on patient needs, the visit may also include an appointment with a Community Health Worker (CHW) for wrap around service referrals such as food pantry, mental health services, and housing. The following questions will be about your experience since that change.

First, how have appointments with the Center differed from the diabetes care you received before September 2020?

Probe: Was this appointment in person or via telehealth?

Probe: Who did you see/meet with that was different from your care before September 2020? Endocrinologist, PCP, CHW, CDCES, pharmacist, clinician, social worker? If so, what did you think of those visits?

Probe: When you saw any of the providers above, how long was your wait?

Probe: What kinds of appointments have you scheduled since then?

1. How did the pace of appointments/meetings feel when you have visited the Center?

Probe: How was the pace of your appointment if it was via telehealth?

1. Since being a Sinai patient, what education have you received to help manage your diabetes?

Probe: Medication and insulin education?

Probe: Dietary/nutrition education?

Probe: Physical activity? Other?

1. How have you applied the education to your diabetes management care routine?
2. Diabetes management involves using new tools, materials and resources to manage your care such as technology to monitor blood glucose levels, nutritional counseling, etc. What kinds of tools did you receive to assist in improving your health?
3. In general, do you find these tools helpful? If you didn’t receive any, which tools would you want the Center to supply?
4. How have you applied the tools and resources you have received from the Center into your diabetes management care routine?

***Patient Engagement Outside of the Center (10 min)?***

1. How do you manage your diabetes outside of the Center?

Probe: How has your diabetes management changed since visiting the Center?

1. When you have questions about your care, who do you contact at the Center?
2. When you have questions or concerns about insurance coverage pertaining to medication and/or other diabetes management resources, who would you contact at the Center for help?
3. How would you go about contacting that individual or team to help you?

***Perceived Center Caregiver Perceptions (15 min)***

1. The Center is a unique model of care that is designed to be patient-centered with a comprehensive team of healthcare professionals to be involved in your healthcare plan, including support with any wrap around services (i.e. transportation, mental health, food pantry) needed. Have you had any of those needs in the last year, if so which ones?

Probe: How has the Center helped addressed these needs?

1. As mentioned before, the Center is designed to have a healthcare team serve your diabetes management needs and care. Who do you consider to be on your healthcare team at the Center?

Probe: Is there a role you’re not seeing on the team that you think is needed?

1. How are you, or are you not, included in developing your healthcare plan?

Probe: Do you feel that your healthcare team hears you? Do you feel heard by your healthcare team?

Probe: Do you feel your feedback is valued by the healthcare team?

Probe: Can you think of an example in which you felt included? Or an example in which you didn't feel included?

1. What do you remember about your experience(s) at the Center that made you feel like you could trust the healthcare team?

Probe: What experience(s) made you feel you could not trust them?

Probe: For example, communicating accurate and relevant care information, boosting your confidence in how you manage your diabetes; or feeling talked over, misled on what medication was accessible to you, etc.

***Wrap-up (5 min)***

1. Is there anything else you’d like to share that we didn’t touch on today?

<<Thank participant.>>
